# Function genomics of abiotic stress tolerance in plants: a CRISPR approach

**DOI:** 10.3389/fpls.2015.00375

**Published:** 2015-05-27

**Authors:** Mukesh Jain

**Affiliations:** Functional and Applied Genomics Laboratory, National Institute of Plant Genome ResearchNew Delhi, India

**Keywords:** abiotic stress, CRISPR-Cas9, genome editing, gene function, transcription

## Introduction

Various abiotic stresses, such as drought, salinity, heat, flooding, ion toxicity and radiation are the major constraints to agricultural production. The understanding of molecular basis of plant response to these environmental conditions has been a major focus of research in the past decades. Several genes/pathways and regulatory networks involved in stress responses have been worked out employing various approaches. Quite a few of these components have been used for engineering abiotic stress tolerance in model and crop plants via classical biotechnological and/or breeding approaches. Success to generate stress-tolerant plants has been achieved to some extent, which has resulted in increased crop yield (Mickelbart et al., 2015). However, novel strategies are desirable to overcome the limitations of classical methods, such as lack of precision and requirement of substantial time to increase the crop production in the current climate change and ever increasing population scenario. The recent availability of genome editing tools provides ample opportunity to introduce targeted modifications in the genome efficiently to study the functional aspects of various components of the genome in diverse plants and offers potential avenues for production of abiotic stress-tolerant crop plants.

Genome editing tools provide a method for introducing targeted mutation, insertion/deletion (indel) and precise sequence modification using customized nucleases in a wide variety of organisms. Zinc finger nucleases (ZFNs), transcriptional activator-like effector nucleases (TALENs) and clustered regularly interspaced short palindromic repeat (CRISPR)-Cas9 (CRISPR-associated nuclease 9) are the most commonly used genome editing tools (Voytas, 2013; Mahfouz et al., 2014; Kumar and Jain, 2015). In general, these sequence-specific nucleases cause double-strand breaks (DSBs) at the target genomic locus/loci, which is/are repaired by the intracellular repair pathways; nonhomologous end joining (NHEJ) or homology-directed repair (HDR). NHEJ leads to the introduction of indels and HDR can be used to introduce specific point mutations or insertion of desired sequences (such as tags or new domains) via recombination. Owing to the simplicity of programming, CRISPR-Cas9 system has opened a plethora of options for genome editing in various biological contexts. Here, we highlight the emerging applications and future avenues of CRISPR-Cas9 system to understand the biology of abiotic stress tolerance in plants.

## CRISPR-Cas9 system: a RNA-guided nuclease for genome engineering

CRISPR-Cas9 system, derived from a prokaryotic RNA-guided defense system (Bhaya et al., 2011), has been most recently developed and is emerging as a method of choice for genome engineering (Harrison et al., 2014; Hsu et al., 2014; Sander and Joung, 2014). Several excellent reviews have described the discovery and mechanism of CRISPR-Cas9 system (Hsu et al., 2014; Sander and Joung, 2014; Kumar and Jain, 2015). The Cas9 nuclease-mediated cleavage is guided by a single guide RNA (sgRNA), which recognizes the target DNA via standard Watson-Crick base pairing (Sander and Joung, 2014; Kumar and Jain, 2015). The existence of a protospacer adjacent motif (PAM; NGG/NAG) site immediately 3′ of the target site is essential. The sgRNAs are of 20–22 nucleotides (nt) in length, which can be easily designed and synthesized as oligonucleotides. Thus, Cas9 nuclease can be targeted to any DNA sequence with 5′-N_(20−22)_-NGG by changing the 20–22 nt guide sequence. Further, the modular nature of CRISPR-Cas9 system, small size of targeting sgRNA, and high efficiency provide additional advantages. The well-designed sgRNAs can provide high specificity with minimal/no off-target effects. Due to these advantages, CRISPR-Cas9 system is amenable to multiplexing, where mutations can be introduced into multiple genes/genomic loci simultaneously. The ease of targeting, high efficiency and possibility of multiplexed modifications with CRISPR-Cas9 system have opened up a broad range of applications in basic and applied research in plant biology.

## Applications of CRISPR-Cas9 in understanding abiotic stress tolerance

**Figure 1:**
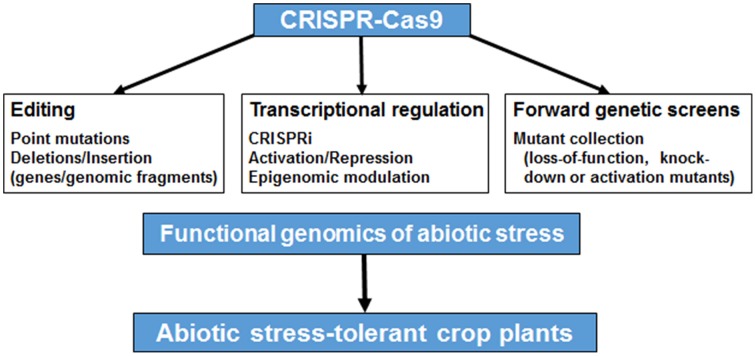
**An overview of the applications of CRISPR-Cas9 system in functional genomics of abiotic stress tolerance**. CRISPR-Cas9 system can be used for genome editing (via introduction of point mutations, insertions or deletions), transcriptional regulation (via CRISPR interference, activation, repression or epigenetic modulation) or forward genetics screens (via generation of loss-of-function, knock-down or activation mutants using sgRNA libraries) for understanding the molecular basis of abiotic stress response, which can lead to generation of abiotic stress-tolerant crop plants.

The use of CRISPR-Cas9 system in engineering abiotic stress tolerance in plants has not been reported yet. CRISPR-Cas9 mediated genome engineering can enable manipulation of nearly any sequence in the genome (limited only by the availability of a PAM site) to reveal its function. CRISPR-Cas9 system has been successfully employed in bacteria, animals and plants for efficient genome editing (Feng et al., 2013; Jiang et al., 2013; Li et al., 2013; Nekrasov et al., 2013; Shan et al., 2013). Several web tools have been developed for designing optimized sgRNA(s) for the target genes/loci to avoid off-target effects (Hsu et al., 2013; Montague et al., 2014). Recently, a web tool CRISPR-P has been developed for designing sgRNAs in more than 20 plant species (Lei et al., 2014). The detailed protocols for targeted mutagenesis in model and crop plants via CRISPR-Cas9 have also been published (Belhaj et al., 2013; Shan et al., 2014). Further, some vectors and a toolkit have been developed for CRISPR-Cas9-mediated plant genome editing (Xing et al., 2014; Kumar and Jain, 2015). Some of these have been made available via Addgene (https://www.addgene.org/crispr/plant/), a non-profit plasmid repository. The availability of these information/resources provides a platform for use of CRISPR-Cas9 system in various applications (editing, transcriptional modulation and genetic screens) to dissect the molecular basis of abiotic stress response and generate stress-tolerant crop plants as outlined in Figure 1.

Abiotic stress is a complex trait, which is governed by multiple genes. There is a substantial interaction between components of several signaling, regulatory and metabolic pathways, which lead to abiotic stress response/adaptation (Nakashima et al., 2009; Hirayama and Shinozaki, 2010; Garg et al., 2014; Mickelbart et al., 2015). Further, plants have undergone whole genome duplication events and a large fraction of genes are represented by multi-gene families with functional redundancy. Many times knock-out of a single gene may not produce any/desired phenotype, thus making it difficult to reveal its function. Due to ease of design and high efficiency of sgRNAs, multiple genes can be targeted simultaneously using CRISPR-Cas9 system, which can overcome the problem posed by functional redundancy of genes. Multiplex genome editing has been successfully implemented in model and crop plants (Li et al., 2013; Mao et al., 2013; Zhou et al., 2014). Such approaches can allow deciphering the role of multiple and functionally redundant genes involved in the same biological process such as abiotic stress response. Another approach could be the pyramiding/stacking of multiple genes involved in a stress response pathway or regulatory network via HDR-mediated gene targeting. The genes involved in stress related gene regulatory network, signal transduction and metabolite production may be targeted via CRISPR-Cas9 technologies for production of stress-tolerant crop plants.

The availability of wild germplasm and genetic variations in crop plants is the key to crop improvement programs. However, the lack of enough natural germplasm, genetic diversity and mutant collections limit both basic and applied research, particularly in crop plants. The genome editing tools provide opportunity to overcome these limitations via creation of such variations in the genome of crop plants. Due to small size and ease of designing, sgRNA libraries targeting almost all the genes can be generated for any plant species, which can be used for generation of genome-scale point mutations and gene knock-outs. The availability of such collections can boost functional genomic studies in crop and non-model plants via large-scale genetic screens. SgRNA libraries can also enable the modification of non-coding genetic elements to facilitate the discovery of gene regulatory regions. Recent reports have demonstrated the potential of CRISPR-Cas9 system to perform robust negative and positive selection screens in human (Shalem et al., 2014; Wang et al., 2014). Although such resources have not been generated in plants as of now, these are expected in near future. The screening of mutants with altered abiotic stress response in plants could enable gene function analyses and generation of stress-tolerant crop varieties.

The use of different versions of Cas9 proteins can further enhance the realm of CRISPR-Cas9 system. For example, dCas9 (catalytically inactive Cas9) can be used to disrupt gene function via CRISPR interference (CRISPRi) (Gilbert et al., 2013; Qi et al., 2013). Some effector domains such as KRAB/SID have been fused with dCas9 to enhance the transcriptional repression (Gilbert et al., 2013; Konermann et al., 2013). The use of paired Cas9 nickases can increase the specificity of mutagenesis substantially (Cho et al., 2014). The fusion of transcriptional activation domains, such as VP16/VP64 to dCas9 can activate the expression of target gene(s) and allow screening for gain-of-function phenotypes for abiotic stress tolerance. A few studies have reported strong activation effects of using multiple sgRNAs for a particular gene promoter (Mali et al., 2013; Perez-Pinera et al., 2013). The use of CRISPR-based synthetic transcriptional activator or repressor to modulate the transcription of target endogenous genes has been demonstrated in plants (Piatek et al., 2015). Tethering of dCas9 to epigenetic modifiers can help defining the role of methylation or different chromatin states in abiotic stress responses/adaptation. The same may be employed to regulate or fine-tune the stress-responsive gene expression. The reassembly of Cas9 protein can generate inducible CRISPR-Cas9 systems to enable spatially precise modifications. The use of light-inducible domains, CIB1 and CRY2, have been successfully demonstrated to construct TALENS (Konermann et al., 2013). The use of conditional (stress-inducible and/or tissue-specific) promoters to drive the expression of Cas9 and sgRNA can avoid undesired pleiotropic effects. A custom-designed zinc finger nuclease along with a heat-shock promoter have been used to induce mutations in an AP2/ERF family transcription factor gene, *ABA-INSENSITIVE 4*, involved in abiotic stress responses (Osakabe et al., 2010). A high frequency (up to 3%) of gene mutations resulting in the desired phenotypes was observed.

A recent study demonstrated the use of CRISPR-Cas9 system in genotyping naturally occurring variations, which allowed distinguishing homozygous biallelic mutants from wild-type (Kim et al., 2014). A few studies have demonstrated the production of homozygous transgenic plants in the first generation (Feng et al., 2013; Mao et al., 2013; Brooks et al., 2014; Zhang et al., 2014; Zhou et al., 2014), which presented the fastest possible method in a crop plant genome modification. Such approaches can reduce breeding or gene transformation time greatly for production of new varieties/transgenic plants with desired traits, such as abiotic stress tolerance. CRISPR technology is being seen as an advancement of plant breeding technologies. Non-transgenic approaches are also available for delivery of such nucleases to produce mutant plants (Marton et al., 2010). As a result, crop varieties produced using these technologies may qualify as non-GM and would have enormous impact on plant biotechnology and breeding.

## Concluding remarks

CRISPR-Cas9 system can greatly facilitate the study of gene/genome function and engineering abiotic stress tolerance in variety of plants. Several studies have demonstrated the robustness and versatility of CRISPR-Cas9 system in different biological contexts. Although the primary application of this tool has been the generation of gene knock-outs so far, harnessing other applications will be very important in the area of stress biology. CRISPR-Cas9 system has not been employed for studying abiotic stress response/adaptation pathways as of now. The development of novel regulatory module(s) from naturally existing components (genes, promoters, *cis*-regulatory elements, small RNAs and epigenetic modifications) can facilitate the engineering of signaling/regulatory and metabolic processes to modulate plant abiotic stress tolerance. Overall, the rapid pace of development and emerging applications of CRISPR-Cas9 system promise its immense contribution in understanding the gene regulatory networks underlying abiotic stress response/adaptation and crop improvement programs to develop stress-tolerant plants.

### Conflict of interest statement

The author declares that the research was conducted in the absence of any commercial or financial relationships that could be construed as a potential conflict of interest.

## References

[B1] Belhaj K. Chaparro-Garcia A. Kamoun S. Nekrasov V. (2013). Plant genome editing made easy: targeted mutagenesis in model and crop plants using the CRISPR/Cas system. Plant Methods 9:39. 10.1186/1746-4811-9-39 24112467 PMC3852272

[B2] Bhaya D. Davison M. Rodolphe B. (2011). CRISPR-Cas systems in bacteria and archaea: versatile small RNAs for adaptive defense and regulation. Ann. Rev. Genet. 45, 273–297. 10.1146/annurev-genet-110410-132430 22060043

[B3] Brooks C. Nekrasov V. Lippman Z. B. Van Eck J. (2014). Efficient gene editing in tomato in the first generation using the clustered regularly interspaced short palindromic repeats/CRISPR-associated9 system. Plant Physiol. 166, 1292–1297. 10.1104/pp.114.247577 25225186 PMC4226363

[B4] Cho S. W. Kim S. Kim Y. Kweon J. Kim H. S. Bae S.. (2014). Analysis of off-target effects of CRISPR/Cas-derived RNA-guided endonucleases and nickases. Genome Res. 24, 132–141. 10.1101/gr.162339.113 24253446 PMC3875854

[B5] Feng Z. Zhang B. Ding W. Liu X. Yang D. L. Wei P.. (2013). Efficient genome editing in plants using a CRISPR/Cas system. Cell Res. 23, 1229–1232. 10.1038/cr.2013.114 23958582 PMC3790235

[B6] Garg R. Verma M. Agrawal S. Shankar R. Majee M. Jain M. (2014). Deep transcriptome sequencing of wild halophyte rice, *Porteresia coarctata*, provides novel insights into the salinity and submergence tolerance factors. DNA Res. 21, 69–84. 10.1093/dnares/dst042 24104396 PMC3925395

[B7] Gilbert L. A. Larson M. H. Morsut L. Liu Z. Brar G. A. Torres S. E.. (2013). CRISPR-mediated modular RNA-guided regulation of transcription in eukaryotes. Cell 154, 442–451. 10.1016/j.cell.2013.06.044 23849981 PMC3770145

[B8] Harrison M. M. Jenkins B. V. O'Connor-Giles K. M. Wildonger J. (2014). A CRISPR view of development. Genes Dev. 28, 1859–1872. 10.1101/gad.248252.114 25184674 PMC4197953

[B9] Hirayama T. Shinozaki K. (2010). Research on plant abiotic stress responses in the post-genome era: past, present and future. Plant J. 61, 1041–1052. 10.1111/j.1365-313X.2010.04124.x 20409277

[B10] Hsu P. D. Lander E. S. Zhang F. (2014). Development and applications of CRISPR-Cas9 for genome engineering. Cell 157, 1262–1278. 10.1016/j.cell.2014.05.010 24906146 PMC4343198

[B10a] Hsu P. D. Scott D. A. Weinstein J. A. Ran F. A. Konermann S. Agarwala V.. (2013). DNA targeting specificity of RNA-guided Cas9 nucleases. Nat. Biotechnol. 31, 827–832. 10.1038/nbt.2647 23873081 PMC3969858

[B11] Jiang W. Zhou H. Bi H. Fromm M. Yang B. Weeks D. P. (2013). Demonstration of CRISPR/Cas9/sgRNA-mediated targeted gene modification in *Arabidopsis*, tobacco, sorghum and rice. Nucl. Acids Res. 41, e188. 10.1093/nar/gkt780 23999092 PMC3814374

[B12] Kim J. M. Kim D. Kim S. Kim J. S. (2014). Genotyping with CRISPR-Cas-derived RNA-guided endonucleases. Nat. Commun. 5, 3157. 10.1038/ncomms4157 24445736

[B13] Konermann S. Brigham M. D. Trevino A. E. Hsu P. D. Heidenreich M. Cong L.. (2013). Optical control of mammalian endogenous transcription and epigenetic states. Nature 500, 472–476. 10.1038/nature12466 23877069 PMC3856241

[B14] Kumar V. Jain M. (2015). The CRISPR-Cas system for plant genome editing: advances and opportunities. J. Exp. Bot. 66, 47–57. 10.1093/jxb/eru429 25371501

[B15] Lei Y. Lu L. Liu H. Y. Li S. Xing F. Chen L. L. (2014). CRISPR-P: a web tool for synthetic single-guide RNA design of CRISPR-system in plants. Mol. Plant 7, 1494–1496. 10.1093/mp/ssu044 24719468

[B16] Li J. Norville J. E. Aach J. McCormack M. Zhang D. Bush J.. (2013). Multiplex and homologous recombination-mediated genome editing in *Arabidopsis* and *Nicotiana benthamiana* using guide RNA and Cas9. Nat. Biotechnol. 31, 688–691. 10.1038/nbt.2654 23929339 PMC4078740

[B17] Mahfouz M. M. Piatek A. Stewart C. N. Jr. (2014). Genome engineering via TALENs and CRISPR/Cas9 systems: challenges and perspectives. Plant Biotechnol. J. 12, 1006–1014. 10.1111/pbi.12256 25250853

[B18] Mali P. Yang L. Esvelt K. M. Aach J. Guell M. Dicarlo J. E.. (2013). RNA-guided human genome engineering via Cas9. Science 339, 823–826. 10.1126/science.1232033 23287722 PMC3712628

[B19] Mao Y. Zhang H. Xu N. Zhang B. Gou F. Zhu J. K. (2013). Application of the CRISPR-Cas system for efficient genome engineering in plants. Mol. Plant 6, 2008–2011. 10.1093/mp/sst121 23963532 PMC3916745

[B20] Marton I. Zuker A. Shklarman E. Zeevi V. Tovkach A. Roffe S.. (2010). Nontransgenic genome modification in plant cells. Plant Physiol. 154, 1079–1087. 10.1104/pp.110.164806 20876340 PMC2971589

[B21] Mickelbart M. V. Hasegawa P. M. Bailey-Serres J. (2015). Genetic mechanisms of abiotic stress tolerance that translate to crop yield stability. Nat. Rev. Genet. 16, 237–251. 10.1038/nrg3901 25752530

[B22] Montague T. G. Cruz J. M. Gagnon J. A. Church G. M. Valen E. (2014). CHOPCHOP: a CRISPR/Cas9 and TALEN web tool for genome editing. Nucl. Acids Res. 42, W401–W407. 10.1093/nar/gku410 24861617 PMC4086086

[B23] Nakashima K. Ito Y. Yamaguchi-Shinozaki K. (2009). Transcriptional regulatory networks in response to abiotic stresses in Arabidopsis and grasses. Plant Physiol. 149, 88–95. 10.1104/pp.108.129791 19126699 PMC2613698

[B24] Nekrasov V. Staskawicz B. Weigel D. Jones J. D. Kamoun S. (2013). Targeted mutagenesis in the model plant *Nicotiana benthamiana* using Cas9-guided endonuclease. Nat. Biotechnol. 31, 691–693. 10.1038/nbt.2655 23929340

[B25] Osakabe K. Osakabe Y. Toki S. (2010). Site-directed mutagenesis in Arabidopsis using custom-designed zinc finger nucleases. Proc. Natl. Acad. Sci. U.S.A. 107, 12034–12039. 10.1073/pnas.1000234107 20508151 PMC2900650

[B26] Perez-Pinera P. Kocak D. D. Vockley C. M. Adler A. F. Kabadi A. M. Polstein L. R.. (2013). RNA-guided gene activation by CRISPR-Cas9-based transcription factors. Nat. Methods 10, 973–976. 10.1038/nmeth.2600 23892895 PMC3911785

[B27] Piatek A. Ali Z. Baazim H. Li L. Abulfaraj A. Al-Shareef S.. (2015). RNA-guided transcriptional regulation in planta via synthetic dCas9-based transcription factors. Plant Biotechnol. J. 13, 578–589. 10.1111/pbi.12284 25400128

[B28] Qi L. S. Larson M. H. Gilbert L. A. Doudna J. A. Weissman J. S. Arkin A. P.. (2013). Repurposing CRISPR as an RNA-guided platform for sequence-specific control of gene expression. Cell 152, 1173–1183. 10.1016/j.cell.2013.02.022 23452860 PMC3664290

[B29] Sander J. D. Joung J. K. (2014). CRISPR-Cas systems for editing, regulating and targeting genomes. Nat. Biotechnol. 32, 347–155. 10.1038/nbt.2842 24584096 PMC4022601

[B30] Shalem O. Sanjana N. E. Hartenian E. Shi X. Scott D. A. Mikkelsen T. S.. (2014). Genome-scale CRISPR-Cas9 knockout screening in human cells. Science 343, 84–87. 10.1126/science.1247005 24336571 PMC4089965

[B31] Shan Q. Wang Y. Li J. Gao C. (2014). Genome editing in rice and wheat using the CRISPR/Cas system. Nat. Protoc. 9, 2395–2410. 10.1038/nprot.2014.157 25232936

[B32] Shan Q. Wang Y. Li J. Zhang Y. Chen K. Liang Z.. (2013). Targeted genome modification of crop plants using a CRISPR-Cas system. Nat. Biotechnol. 31, 686–688. 10.1038/nbt.2650 23929338

[B33] Voytas D. F. (2013). Plant genome engineering with sequence-specific nucleases. Ann. Rev. Plant Biol. 64, 327–350. 10.1146/annurev-arplant-042811-105552 23451779

[B34] Wang T. Wei J. J. Sabatini D. M. Lander E. S. (2014). Genetic screens in human cells using the CRISPR-Cas9 system. Science 343, 80–84. 10.1126/science.1246981 24336569 PMC3972032

[B35] Xing H. L. Dong L. Wang Z. P. Zhang H. Y. Han C. Y. Liu B.. (2014). A CRISPR/Cas9 toolkit for multiplex genome editing in plants. BMC Plant Biol. 14:327. 10.1186/s12870-014-0327-y 25432517 PMC4262988

[B36] Zhang H. Zhang J. Wei P. Zhang B. Gou F. Feng Z.. (2014). The CRISPR/Cas9 system produces specific and homozygous targeted gene editing in rice in one generation. Plant Biotechnol. J. 12, 797–807. 10.1111/pbi.12200 24854982

[B37] Zhou H. Liu B. Weeks D. P. Spalding M. H. Yang B. (2014). Large chromosomal deletions and heritable small genetic changes induced by CRISPR/Cas9 in rice. Nucl. Acids Res. 42, 10903–10914. 10.1093/nar/gku806 25200087 PMC4176183

